# Super-Resolution Imaging with Graphene

**DOI:** 10.3390/bios11090307

**Published:** 2021-08-30

**Authors:** Xiaoxiao Jiang, Lu Kong, Yu Ying, Qiongchan Gu, Jiangtao Lv, Zhigao Dai, Guangyuan Si

**Affiliations:** 1 College of Information Science and Engineering, Northeastern University, Shenyang 110004, China; jxx316@mail.ustc.edu.cn (X.J.); 1801889@stu.neu.edu.cn (L.K.); guqiongchan@neuq.edu.cn (Q.G.); lvjiangtao@neuq.edu.cn (J.L.); 2 College of Information and Control Engineering, Shenyang Jianzhu University, Shenyang 110168, China; yingyu@sjzu.edu.cn; 3 Engineering Research Center of Nano-Geomaterials of Ministry of Education, Faculty of Materials Science and Chemistry, China University of Geosciences, 388 Lumo Road, Wuhan 430074, China; daizhigao@cug.edu.cn; 4 Melbourne Centre for Nanofabrication, Victorian Node of the Australian National Fabrication Facility, Clayton, VIC 3168, Australia

**Keywords:** super-resolution imaging, graphene plasmonics, evanescent-field enhancement

## Abstract

Super-resolution optical imaging is a consistent research hotspot for promoting studies in nanotechnology and biotechnology due to its capability of overcoming the diffraction limit, which is an intrinsic obstacle in pursuing higher resolution for conventional microscopy techniques. In the past few decades, a great number of techniques in this research domain have been theoretically proposed and experimentally demonstrated. Graphene, a special two-dimensional material, has become the most meritorious candidate and attracted incredible attention in high-resolution imaging domain due to its distinctive properties. In this article, the working principle of graphene-assisted imaging devices is summarized, and recent advances of super-resolution optical imaging based on graphene are reviewed for both near-field and far-field applications.

## 1. Introduction

Graphene is a two-dimensional material made up of sp^2^-hybridized carbon arranged in a honeycomb crystal lattice with one-atom thickness [1]. Since single-layer graphene flakes were experimentally isolated by Geim and Novoselov in 2004 [2], it has drawn remarkable attention owing to its perfect structural [3], optical [4], electric [5], and thermal [6,7] properties. In the past few years, the research on graphene has made significant progress because many new effective synthesis methods of graphene in different types have been explored and accomplished including micromechanical exfoliation [8], growth on various substrates [9], deposition [10,11,12], and so on [13,14,15].

One of the most important properties of graphene is that the complex conductivity can be dynamically tuned by external parameters [16,17,18] such as electric field, magnetic field, and gate voltage [19,20], which makes graphene behave like thin metallic materials that possess a negative permittivity at low frequencies [21]. In addition, the surface plasmon polariton in graphene is different from conventional plasmons in both metals and two-dimensional electron gases [22,23]. For instance, graphene plasmons show high confinement and relatively low loss with more flexible features. All these unique features have made graphene a promising candidate for a variety of crucial applications [24,25], such as super-resolution imaging and optical biosensing [26,27,28].

After the first microscope was invented and named as well as applied to observe cells successfully in the 17th century, microscopic techniques remain the most widespread imaging method and always play an irreplaceable role in the research field of nanotechnology and biotechnology, especially in bioscience [29], owing to its numerous advantages (e.g., noninvasive, reliable, suitable for various samples, and so on). However, the resolution of traditional fluorescence microscopy is fundamentally limited to *λ⁄2NA* (*λ* is the wavelength of the incident light and *NA* is the numerical aperture). This intrinsic limit, also known as diffraction limit [29,30], has become the main obstacle to high-resolution optical imaging. Thus far, a great number of novel methods for achieving super-resolution imaging have been proposed and demonstrated experimentally in both near field and far field, such as near-field scanning optical microscopy (NSOM) [31,32,33]; far-field superlens (FSL) [34,35,36], hyperlens [37,38,39], and metalens [40,41,42,43]; stimulated emission depletion microscopy (STED) [44,45,46,47]; stochastic optical reconstruction microscopy (STORM) [48,49,50,51,52]; structured illumination microscopy (SIM) [53,54,55]; plasmonic structured illumination microscopy derived from SIM [56,57,58], and so on [59,60]. It is worth mentioning that graphene-related materials exhibit different properties according to their lateral size, number of layers and oxidation degree. For specific applications, graphene of different lateral sizes shows different performance expressions even if they are similar in terms of defects and number of layers. As the size increases, it becomes more difficult to disperse and composite with graphene. These controllable properties will also affect the device performance of graphene-assisted imaging components when graphene is integrated into a super-resolution imaging system. However, remarkable improvements are achievable. There are several super-resolution technologies which have been cooperated with graphene such as NSOM, hyperlens, superlens, STORM, and SIM. This review presents a comprehensive summary of the research on super-resolution optical imaging based on graphene, including both experimental and theoretical studies. The article first introduces the principle of super-resolution imaging with graphene and the following sections will focus on different imaging methods with graphene found in the bibliography and categorized by application regions (near field and far field).

## 2. Working Principle of a Super-Resolution Imaging System with Graphene

Achieving super-resolution imaging means to overcome the diffraction limit originated from the exponential decay of the evanescent waves which carry the high spatial frequency information of the objects. In essence, overcoming the fundamental limit means magnifying the evanescent waves directly or converting evanescent waves to propagating ones and further providing magnification. Graphene can significantly enhance evanescent fields due to the fact that its conductivity can be tuned in the infrared and terahertz (THz) regions [61,62].

Among all these amazing properties of graphene, the one that is most worthy of mention is that the surface conductivity of graphene can be tuned via external parameters [19,63]. The surface conductivity of graphene can be calculated by the Kubo formula [64,65,66]:

(1) $$\sigma_{g}=\frac{ie^{2}k_{B}T}{\pi \hbar^{2}\left(w+i/\tau \right)}\left(\frac{\mu_{c}}{k_{B}T}+2\ln\left(e^{-\frac{\mu_{c}}{k_{B}T}}+1\right)\right)+\frac{ie^{2}}{4\pi \hbar }ln\left[\frac{2\mu_{c}-\hbar \left(w+i/\tau \right)}{2\mu_{c}+\hbar \left(w+i/\tau \right)}\right]$$

where $e=-1.6\times 10^{-19}C$ is the electron charge, $k_{B}=1.3806505\times 10^{-23}\;J/K$ is Boltzmann constant, and $\hbar =1.05\times 10^{-34}\;J\cdot s$ is the reduced Planck constant. The surface conductivity of graphene depends on Kelvin temperature *T*, the radian frequency *w*, the momentum relaxation time *τ*, and the chemical potential $\mu_{c}$. The chemical potential $\mu_{c}$ depends on the carrier density and can be controlled by gate voltage, electric field, magnetic field, and doping [67,68]. The effective optical permittivity of graphene can be written as:

(2) $$\varepsilon_{g}=1+\frac{i\sigma_{g}\eta_{0}}{k_{0}\Delta }$$

where $\eta_{0}\approx 377\;\Omega$ is the impedance of air and Δ is the thickness of graphene. It should be noted that the permittivity of graphene depends on the surface conductivity. At low frequencies such as infrared and THz range, graphene behaves like a thin metal layer with negative permittivity because the imaginary part of conductivity can be tuned to be positive via an external parameter. Due to its capability of enhancing the evanescent field, graphene has become a promising candidate for imaging applications [69,70,71].

Moreover, since surface plasmons (SPs) have been experimentally demonstrated in graphene [28], graphene plasmons (the coupled state between photons and collective Dirac electrons in graphene) have found extensive practical applications [72,73]. Compared to the SPs excited on metallic surfaces, the field of SPs supported by graphene possesses significant advantages. Graphene plasmons are more tightly confined on the surface of graphene with an effective index capable of reaching 70 in the far-infrared region, compared to the index value of 1.03 for SPs on metal surfaces [4]. Besides, the damping loss of graphene plasmons is relatively low and the propagation distance could reach dozens of wavelengths of SPs [74]. It is also important that the SPs excited on graphene can be simply manipulated by external parameters [75,76,77]. The abovementioned advantages of graphene plasmons have made graphene a momentous candidate for a variety of practical applications [78,79,80], especially in the super-resolution imaging field [81,82,83]. Most recently, real-space imaging of acoustic plasmons in large-area graphene was experimentally demonstrated, enabling a new platform for strong light–matter interaction [84]. In addition, more graphene quantum dot based materials have been widely used for sensing and bio-imaging applications [85].

## 3. Super-Resolution Imaging Cooperated with Graphene

### 3.1. Graphene-Assisted Super-Resolution Imaging in Near Field

**Figure 1:**
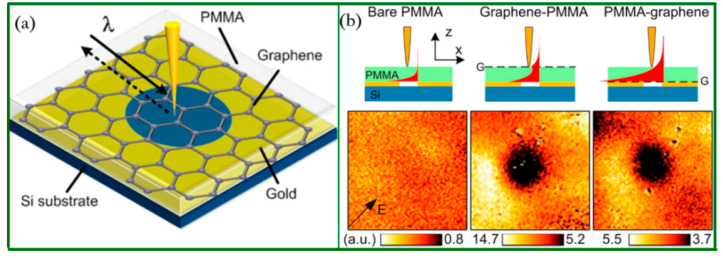
Graphene-assisted imaging. (**a**) Schematic drawing of the graphene–PMMA hybrid system with a 1.5 μm diameter buried hole for near field imaging. (**b**) Near-field amplitude images collected with a bare PMMA layer, graphene–PMMA combination, and PMMA–graphene combination, respectively. In both graphene-assisted hybrid systems, the subwavelength hole can be clearly resolved. All images are 4 μm × 4 μm. Reprinted with permission from [70]. Copyright 2014 American Chemical Society.

For near-field applications, graphene has been integrated with NSOM, superlens, and wire medium, and therefore can provide magnification of the evanescent waves for achieving super-resolution imaging [86,87,88] via tunable conductivity and the coupling of graphene plasmons [89,90,91]. Monolayer graphene was demonstrated to offer a sevenfold enhancement of evanescent information and successfully resolve buried structures at a 500 nm depth with *λ*⁄11-resolution via graphene-enhanced NSOM in 2014 [70]. A sharp probe, the key component of NSOM imaging systems, was used to pick up the evanescent signals, but it was incapable of imaging buried structures. However, when a monolayer graphene was coated on the top of a polymethyl methacrylate (PMMA) sample, the evanescent information could be enhanced and detected by the probe due to the surface plasmon polaritons of graphene with ultrasmall plasmon wavelengths as shown in Figure 1. In this case, the resolution of this imaging system is mainly determined by the wavelength of graphene plasmon rather than the free-space wavelength. With the assist of a graphene layer, a buried hole can be resolved clearly compared to the bare PMMA sample. Moreover, the configuration is more convenient and feasible than the superlens–NSOM combination, which needs a perfect superlens for practical applications.

**Figure 2:**
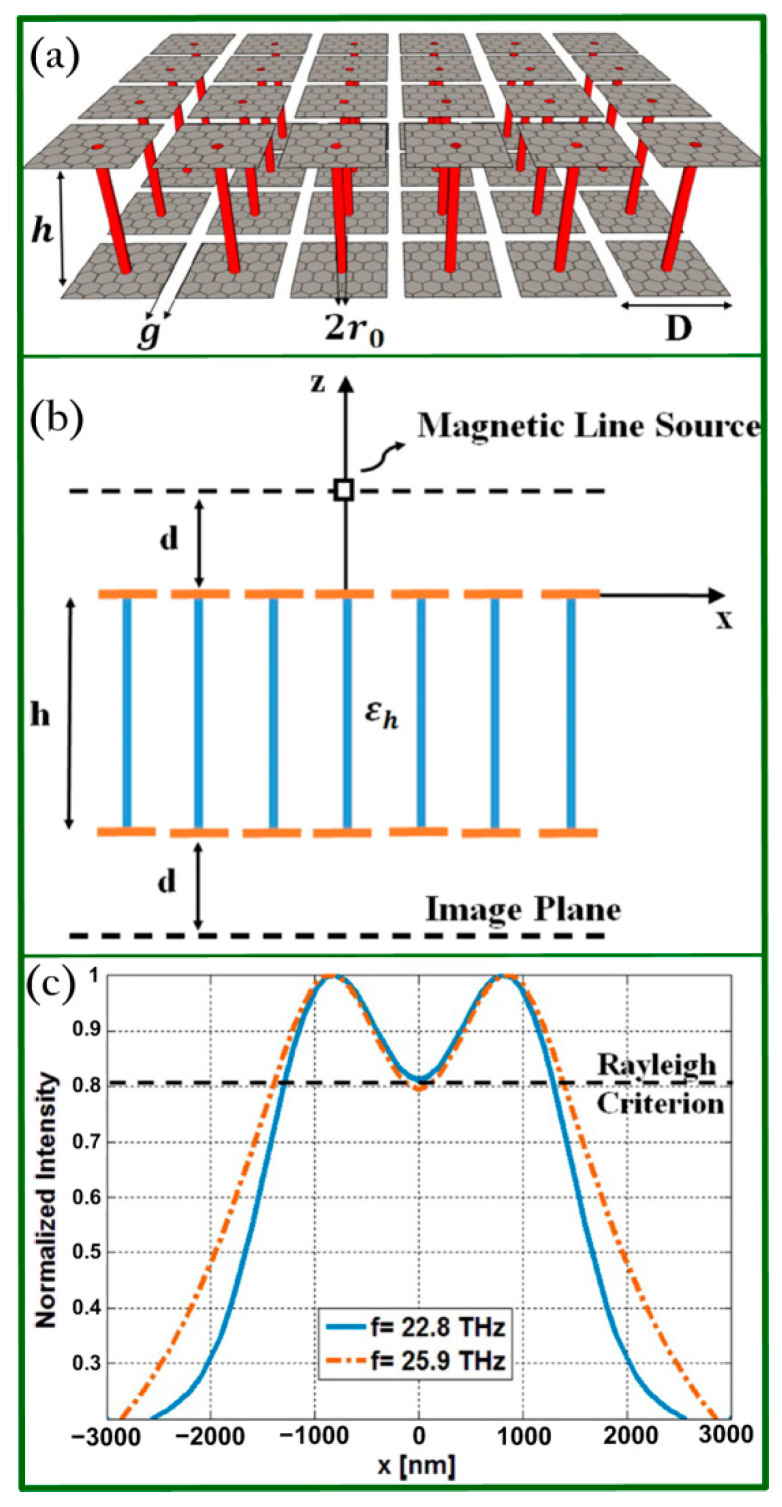
(**a**) Diagram of WM slab-loaded GNMs. (**b**) Sketch of the WM slab-loaded GNMs using a magnetic line source. (**c**) Normalized intensity distribution calculated at the image plane for 22.8 and 25.9 THz. Reprinted with permission from [81]. Copyright 2015 American Institute of Physics.

In 2015, Forouzmand and co-workers proposed two kinds of novel graphene-loaded wire medium (WM) slab structures that were suitable for dual-band or tunable broadband super-resolution imaging in near field [81,82]. Figure 2a schematically illustrates the proposed devices of WM slab loaded graphene nanopatch metasurfaces (GNMs). The principle of the aforementioned structures relies on the enhancement of the evanescent waves due to the coupling of the SPs between the lower and upper graphene as well as the remarkable waveguiding of the evanescent waves of the WM slab [87]. In addition, the performance of the structures was analyzed in the presence of a magnetic line source (Figure 2b). The resolution was quantified by using the half power beam width (HPBW) [85] and the Rayleigh criterion. Super-resolution imaging effects may enable more extensive applications with practical potential and possess the capability of resolving closely spaced light sources, as indicated in Figure 2c using the Rayleigh criterion. The working mechanism of this superlens relies on graphene plasmon resonances and can significantly amplify the evanescent waves that include the high spatial frequency information and restore them at the image plane. Graphene plasmons excited at both lower and upper GNMs can be coupled and further used to help amplify the evanescent waves. As expected, the hybridization-enhanced bilayer design could achieve higher resolution because of stronger interactions than the monolayer graphene design. Note that the number of layers will affect the imaging performance since multilayer designs can involve the interactions between neighboring layers and therefore impact optical properties compared with the monolayer layout.

**Figure 3:**
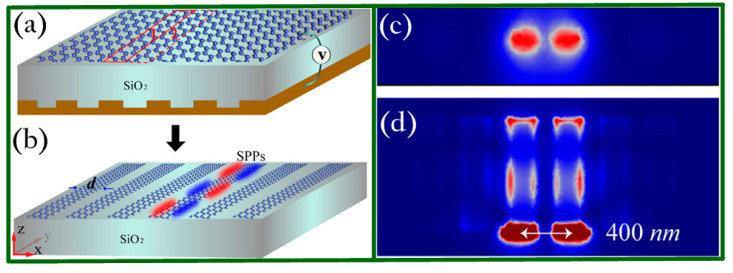
Voltage-gate-assisted hybrid superlens. (**a**) Schematic layout of the proposed device. (**b**) The equivalent model for a certain wavelength. (**c**) Top-view and (**d**) cross-sectional view of the electric field distribution when the two sources are 400 nm spaced. Note that the frequency is 5 THz with λ/150 resolution. Reprinted with permission from [71]. Copyright 2017 Springer Nature.

Another graphene-assisted subwavelength imaging device was reported in 2017 [71] which relied on the Fabry–Perot resonance of graphene edge plasmon waves [88,89] for breaking the diffraction limit in the THz frequency range. The superlens was constituted by a single sheet of graphene and a metallic grating voltage gate. Figure 3a shows the perspective of the proposed superlens and Figure 3b demonstrates the equivalent model for a certain frequency. The most noteworthy advantage of this superlens is that it can be easily manipulated in a wide range from 4.3 THz to 9 THz by adjusting the gate voltage. In addition, one can readily obtain subwavelength targets magnified images by replacing the grating gate with a radial shape. The best resolution achieved was 400 nm as shown in Figure 3c (top-view) and Figure 3d (cross section), leading to great potential applications in THz near field imaging systems. In addition, one should note that the impact of lateral size on device performance is significant since graphene edge plasmons are highly dimension dependent. For instance, by reducing the size of the graphene structures, one can readily manipulate the working frequency of graphene plasmons from longer wavelengths to the visible and near-infrared range.

**Figure 4:**
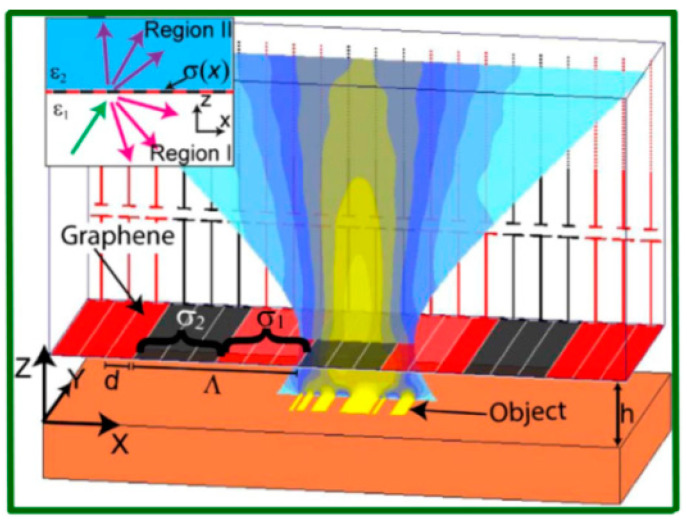
Working mechanism of GNOM. The width of the graphene strips placed side-by-side (in the x-y plane) is represented by *d* and Λ is the periodicity. Reprinted with permission from [72]. Copyright 2017 The Optical Society.

A fast-paced graphene-based near-field optical microscopy (GNOM) for overcoming the diffraction limit was proposed by Inampudi and coworkers who utilized the electronic scanning property of graphene gratings (different from the mechanical scanning of a sharp tip in NSOM) [72]. Figure 4 schematically shows the proposed GNOM. Based on the fact that the graphene’s surface conductivity is reconfigurable, grating scattered light can be collected and then processed by the rigorous coupled-wave analysis. In this work, the authors demonstrated the highest resolution of λ⁄16 (λ = 10 μm) theoretically. In addition, numerical optimization based on the genetic algorithm was also demonstrated to design an optimum set of diffraction grating and minimize the artifacts in the image, which was an extremely challenging task [90].

**Figure 5:**
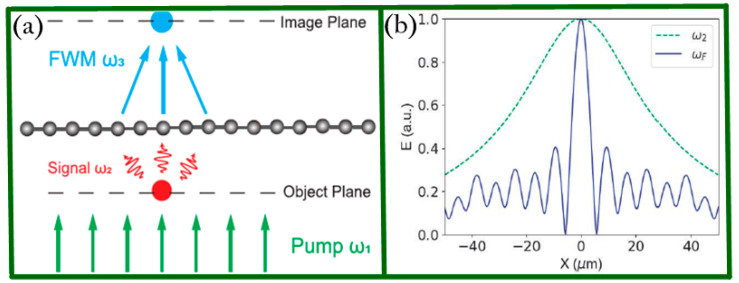
(**a**) Schematic drawing of the negative reflection lens based on FWM process. (**b**) Electric field for the signal and FWM waves at the imaging plane. Reprinted with permission from [73]. Copyright 2018 The Optical Society.

In 2018, Liu and co-workers introduced a graphene sheet as an ultrathin nonlinear negative reflection lens for achieving super-resolution imaging based on four wave mixing (FWM) process in the terahertz regime [73] thanks to the fact that graphene possesses strong nonlinear electromagnetic response. This is totally different from traditional materials which reduce the field strength necessary for the nonlinear process, such as FWM [89]. Figure 5a schematically demonstrates the working principles. It has been theoretically predicted and experimentally validated that the FWM wave can be focused on a point in the image plane via modulating the incident angle. Figure 5b shows the electric field of the signal waves without the graphene lens (dotted line). As a comparison, FWM waves are plotted using the solid line, and one can see that the full width at half-maximum obtained is 3.28 μm (λ⁄5), which indicates great potential applications in THz microscopy.

### 3.2. Graphene-Assisted Super-Resolution Imaging in Far Field

**Figure 6:**
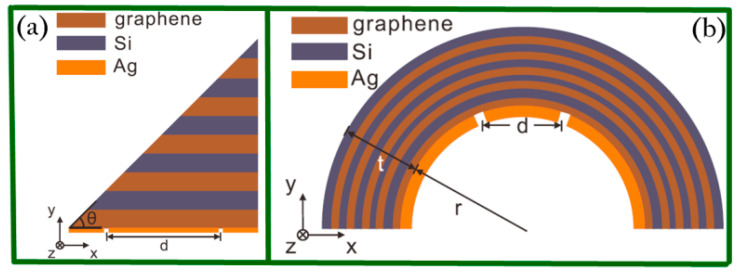
(**a**) Triangle-shaped hyperlens. The layered structures are covered with a silver coating (100 nm thick) with two slits (10 nm width) separated by d = 3.3 μm. (**b**) Cylindrical hyperlens is coated with a silver layer (100 nm thick) with two slits (50 nm wide) separated by d = 1 μm in the inner surface whose radius is defined as r = 1 μm. Reprinted with permission from [69]. Copyright 2013 The Optical Society.

In far field, graphene has normally been integrated with a hyperlens. Surface plasmons can help convert the evanescent waves to the propagating ones and therefore provide magnification for achieving super-resolution imaging via the tunable conductivity and the coupling of graphene plasmons. In 2013, Zhang and coworkers designed two kinds of different hyperlenses [69] composed by alternating graphene/dielectric layered structures for achieving super-resolution optical imaging in the mid-infrared range. They were triangle-shaped and cylindrical hyperlenses, respectively. Figure 6 schematically shows the cross-section view of the proposed novel layered structures.

**Figure 7:**
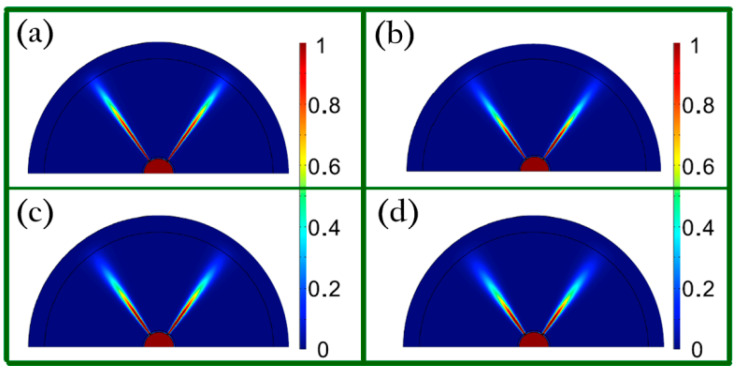
Far-field sub-diffraction imaging results under different conditions: (**a**) λ = 9.2 µm, *µ_c_* = 0.0965 eV, ε_||_ = 0.1419 + 0.2933 *i*, ε_┴_ = −159.8 + 378.6 *i*; (**b**) λ = 10.2 µm, *µ_c_* = 0.085 eV, ε_||_ = 0.1058 + 0.3709 *i*, ε_┴_ = −74.07 + 341.7 *i*; (**c**) λ = 11.2 µm, *µ_c_* = 0.075 eV, ε_||_ = 0.4481 + 0.4678 *i*, ε_┴_ = −122.9 + 152.8 *i*; (**d**) λ = 12.2 µm, *µ_c_* = 0.067 eV, ε_||_ = 0.761 + 0.5831 *i*, ε_┴_ = −90.05 + 86.94 *i*. Reprinted with permission from [69]. Copyright 2013 The Optical Society.

The working mechanism is identical for these two kinds of hyperlenses, which relies on the fact that the hyperbolic dispersion curve can amplify and support the propagation of the evanescent waves straightly along the normal direction of the layered structures and form two imaging spots at the output plane under the condition of Re(ε∥)>0, Re(ε⊥)<0, and Re$\left(\varepsilon_{∥}\right)$→0 ($\varepsilon_{∥}\;,\;\varepsilon_{⊥}$ are the permittivity of the structure along tangential and radial directions, respectively). The distances between two imaging spots are *d⁄cosθ* and *d(r + t))⁄r* for the triangle-shaped and cylindrical hyperlenses, respectively. In addition, it is worth noting that the layered structures can achieve propagation of the evanescent waves for a fixed wavelength by manipulating *μ_c_*, as shown in Figure 7, enabling extensive potential applications in broadband super-resolution imaging.

**Figure 8:**
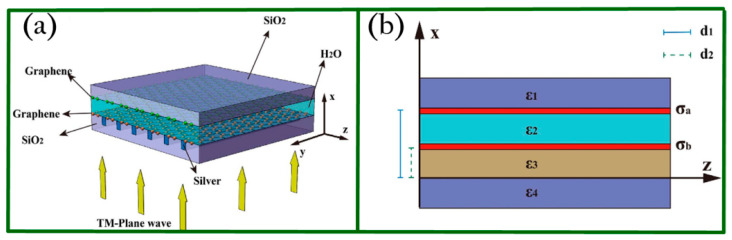
(**a**) Layout and working principle of GNMS. Two layers of graphene form the nanocavity, which is filled with water. (**b**) Cross section of the GNMS. Reprinted with permission from [83]. Copyright 2016 Springer Nature.

In 2016, Yang and coworkers proposed a graphene nanocavity on metasurface structure (GNMS) [83] to excite graphene surface plasmons at mid-infrared waveband and achieved super-resolution optical imaging by integrating the GNMS device with plasmonic structured illumination microscopy (PSIM). Figure 8a shows the schematic of GNMS and one can see that two layers of graphene are involved to form a cavity filled with water. Figure 8b illustrates the cross section of GNMS.

**Figure 9:**
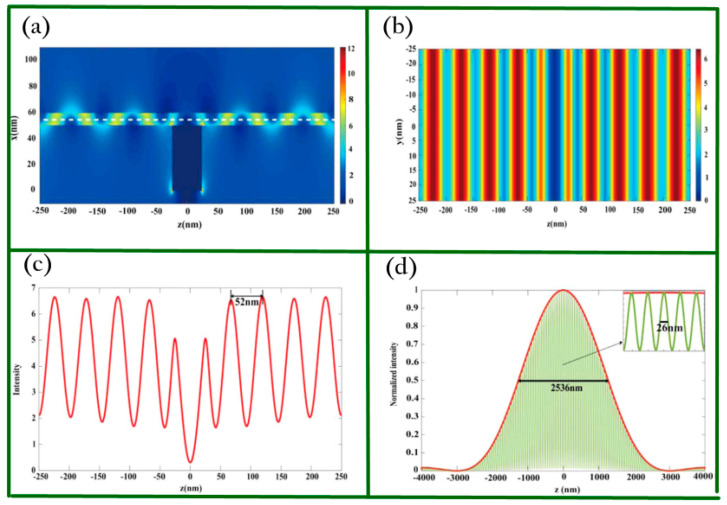
Imaging performance of the nanocavity-enhanced metasurface device. (**a**) Side view of electric field mapping. (**b**) Top view mapping. (**c**) Intensity plot of the white dashed line shown in (**a**). (**d**) Normalized intensity of normal epifluorescence microscopy (red) and GNMS-assisted PSIM (green). Reprinted with permission from [83]. Copyright 2016 Springer Nature.

To further study the property of the GNMS device, the function of the grating and the effect of the structural parameters on the graphene plasmonic interference pattern were discussed. According to the results, one can learn that the period of the graphene plasmonic interference pattern is 52 nm when the wavelength of the incident light is 7 μm. When the GNMS device is integrated with PSIM, an imaging resolution of 26 nm can be achieved because of the graphene plasmons with deep sub-wavelength. Figure 9a–c shows the simulation results of the electric distribution of graphene plasmonic interference patterns. Figure 9d presents the performance of GNMS quantified by using the full width at half maximum (FWHM) of the point spread function (PSF). Since mid-infrared is safe for biological cells, this work may pave a new way for optical super-resolution imaging at mid-infrared waveband for biological research.

**Figure 10:**
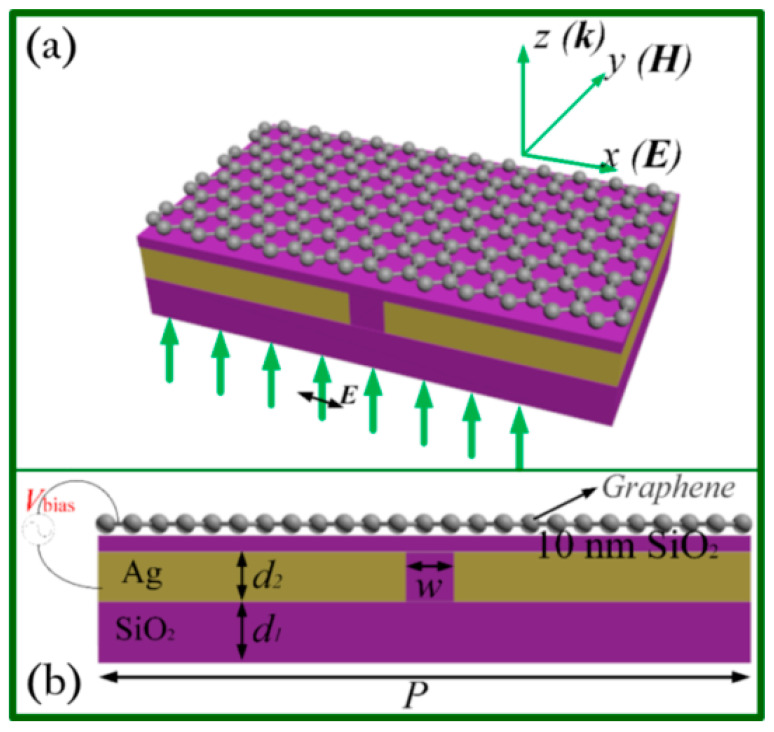
The schematic diagram of the GMS design. (**a**) The perspective view with coordinates and (**b**) the cross-sectional layout. *V_bias_* is the control voltage. The thickness of the flat SiO_2_ substrate is *d_1_* = 200 nm. A thin Ag film with *d_2_* = 50 nm thickness is covered by a 10 nm thick SiO_2_ film. A nanoslit with *w* = 30 nm width in x direction and an infinite length in y direction is filled with SiO_2_ in the Ag film. The unite cell has a same periodicity of *P* = 350 nm in both x and y direction. Reprinted with permission from [86]. Copyright 2017 The Optical Society.

Moreover, the same group proposed another elegant design to realize wide-field optical imaging based on a hybrid graphene on metasurface structure (GMS) model [86]. Figure 10a is the schematic view of GMS, including a monolayer graphene deposited on a SiO_2_/Ag/SiO_2_ multilayer design. From Figure 10b, one can see more clearly the cross-sectional view of a unite cell of the GMS.

**Figure 11:**
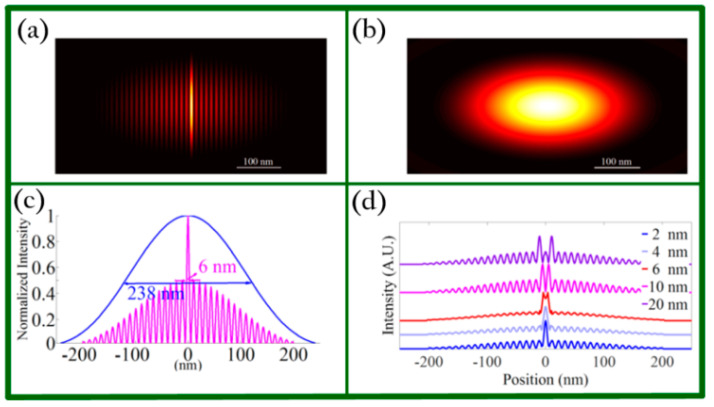
(**a**) The reconstructed image of a point object in the x direction. (**b**) The image of the point object in the conventional fluorescence microscopy system. (**c**) FWHF comparison between (**a**) (red line) and (**b**) (blue line). (**d**) Illustration resolving capability of GMS–PSIM system of two point objects separated with different distances of 2, 4, 6, 10, and 20 nm. Reprinted with permission from [86]. Copyright 2017 The Optical Society.

In this work, it is crucial to utilize the most significant feature of graphene plasmons, which is the ultra-high wave vector, to combine the model of GMS with the PSIM method and further achieve super-resolution imaging. The authors employed the finite-difference time-domain (FDTD) method to model and simulate the GMS structure and found that the standing wave of surface plasmons (SW-SPs) with an 11 nm period can be achieved on graphene. Furthermore, when the GMS structure was applied in the PSIM method, they found that an imaging resolution of 6 nm could be obtained for a 980 nm illumination wavelength which was improved 39.6-fold in comparison with the conventional microscopy technique with resolution of 283 nm. Additionally, the resolving capability of GMS–PSIM system was acquired by imaging two point objects separated by 6 nm. The simulation results are shown in Figure 11.

In addition to the tunable surface conductivity and graphene plasmons, graphene has been introduced to the applications of electron microscopy to achieve super-resolution imaging of wet cells by utilizing the impermeable and conductive properties. More important bioimaging applications can be enabled using graphene-based nanomaterials (e.g., graphene quantum dots) and their derivatives [92,93,94]. Wojcik and coworkers utilized graphene which was synthesized by chemical vapor deposition (CVD) as an impermeable and conductive membrane to enable electron microscopy of wet and untreated cells, enabling direct electron microscopy of wet cells via simple sample preparation without demanding special devices and equipment as well as the comparable contrast and resolution with a conventional scanning electron microscope [95]. To summarize the above discussion on progress in super-resolution imaging with graphene-based nanostructures in infrared and THz frequencies for both near field and far field, Table 1 lists more detailed information for a more comprehensive and systemic comparison. Note that most of the experimentally demonstrated applications so far have been limited to infrared and THz frequencies and it is extremely challenging to further extend the working ranges due to the intrinsic properties of graphene plasmons. However, more flexible and tunable devices are desired to meet the increasing demands of practical applications. In addition, more efforts should be made to further reduce the fabrication cost of such devices to enable massive production.

## 4. Alternative Imaging Devices Integrated with Graphene Oxide

**Figure 12:**
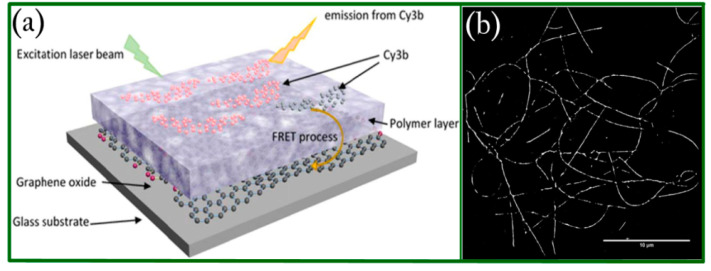
(**a**) The schematic of the stacked films to perform qSTORM with GO coating. Note that the Cy3b fluorophores belong to the Cyanine family. (**b**) Reconstructed STORM image of peptide fibers. Reprinted with permission from [102]. Copyright 2018 Springer Nature.

Graphene oxide (GO) has also found extensive applications in integrated photonics [96,97] and therapy [98,99,100,101]. Moreover, it can be used to cooperate with quenched stochastic optical reconstruction microscopy (qSTORM) to achieve the super-resolution imaging effect of self-assembled peptide fibrils and Escherichia coli. In 2018, Li et al. experimentally demonstrated that GO coating with qSTORM could increase the imaging resolution and contrast [102] when imaging the peptides and bacteria via the strong quenching effect. Due to the same hexagonal lattice and similar electronic properties for both graphene and GO, the quenching effect is advisable with GO. In the experiments the authors designed, the stacked films were employed to perform qSTORM with GO coating as illustrated in Figure 12a. In this design, the GO coating played a significant role in removing background noise and improving the signal-to-noise ratio. Figure 12b shows the reconstructed STORM image of peptide fibers. In the imaging performance of the self-assembled peptide fibrils, the contrast changed from 13 (±47%) to 133 (±40%) with GO and the resolution of the image with GO and without GO was 19 nm and 23 nm, respectively, using the Fourier Ring Correlation method [103,104] with qSTORM. In the imaging performance of Escherichia coli, the contrast changed from 24 (±27%) to 3317 (±37%) with GO. The resolution of imaging peptide fibrils and Escherichia coli was 11 nm and 24 nm with 5 nm GO coating by using the feature of interest (FOI) metric, leading to a dramatic improvement. Note that the oxidation grade of GO can affect the imaging quality since the carbon-oxygen ratio can simply modulate the photoluminescence quenching capabilities and therefore further control the device performance.

## 5. Conclusions and Outlook

To conclude the review, the working principles, implementations, and performances of the super-resolution imaging integrated with graphene/GO have been comprehensively summarized. Due to the most useful property that the surface conductivity of graphene can be rapidly tuned by external parameters, graphene is capable of enhancing the evanescent waves. The present review strongly suggests that graphene has great advantages in super-resolution imaging in the infrared and THz regions both in near field and far field. However, the use of the graphene for super-resolution imaging is still at a very early stage, which means that we still need a lot of time and efforts to explore its full potential experimentally. In our opinion, thinner, faster, and more reliable imaging devices using graphene-assisted components are highly desired for various practical applications and therefore, more efforts should be made to develop related devices. More flexible components are also needed to meet the increasing future demands. Moreover, thorough investigations on structural parameters are necessary for studying their impact and working mechanisms. Given the progress in both the theory and fabrication of the graphene-assisted materials, there is no doubt that more methods and devices on super-resolution imaging with graphene will be developed to meet the increasing demanding of more practical application requirements in the near future.

## Figures and Tables

**Table 1:** Summary of super-resolution imaging systems integrated with graphene.

Year	Device Type	Near/Far Field	Waveband	Resolution	Physical Mechanism	Refs.
2013	Hyperlens	Far field	Mid-infrared	λ/10	Tunable conductivity	[69]
2014	SNOM	Near field	Infrared	λ/11	Graphene plasmonic	[70]
2015	Superlens	Near field	60 THz	λ/50	Graphene plasmonic	[80]
2015	Wire medium	Near field	22.8 THz 25.9 THz	λ/10 0.14λ	Graphene plasmonic	[81]
2015	Wire medium	Near field	THz	λ/10	Graphene plasmonic	[82]
2016	GNMS	Far field	Mid infrared	26 nm	Graphene plasmonic	[83]
2017	Superlens	Near field	4.3~9 THz	400 nm	Tunable conductivity	[71]
2017	GNOM	Near field	30 THz	λ/16	Tunable conductivity	[72]
2017	GMS	Far field	Infrared	6 nm	Graphene plasmonic	[86]
2018	FWM	Near field	THz	λ/5	Tunable conductivity	[73]
